# Supplementary material for: Structural and Histomorphological Evaluation of the Stifle Joint Capsule in Canine Congenital Patellar Luxation and Cranial Cruciate Ligament Rupture
Source: Animals (Basel). 2025 Dec 9;15(24):3545. doi: 10.3390/ani15243545 (PMC12730020; doi:10.3390/ani15243545)
Supplement: Supplementary file 1 [file animals-15-03545-s001.zip › animals-3982353-supplementary.pdf]

Table 1- Singleton Classification for patellar luxation, including tibial tuberosity deviation degrees

**Grade**	**Description**	**Tibial Tuberosity Deviation**
I	Intermittent luxation with the patella returning to its normal position spontaneously.	Slight deviation
II	Frequent luxation where the patella can be manually reduced, but it luxates intermittently on its own.	Deviation of up to 30°
III	Persistent luxation where the patella remains out of the trochlear groove but can be manually reduced.	Deviation of 30° to 60°
IV	Permanent luxation with the patella fixed outside the trochlear groove and severe skeletal deformities.	Deviation of 60° to 90°

Table 2- Step-by-step process for the preparation of slides for histological staining, ensuring consistent and high-quality tissue samples for analysis. Paraplast®, Fa. Shandon Labortechnik GmbH, Frankfurt, Germany. Jung Histoslide 2000R®, Leica Instruments GmbH, Nussloch, Germany. HistoBond®, Marienfeld, Lauda-Königshofen, Germany. Eurocassette®, Fa. Reichert-Jung, Heidelberg, Germany.

Step	Process	Details
1. Sample Collection	Surgical or post-mortem	Laterally parapatellar, 0.5-2 cm^2 size with scalpel (Aesculap®, USA). Attention was paid to the vastus lateralis muscle, which extends proximally into the joint capsule. The primary considerations for this biopsy location were to ensure good conditions for the healing process and to tighten the joint capsule (Schebitz H and Brass 1999)
2. Fixation	4% formaldehyde solution	55 to 60 hours at +4°C. The fixative volume was 20 times that of the sample to ensure complete penetration. Accordingly, large containers were used to allow the joint capsule biopsies to float freely. For this study, tissue samples were fixed for approximately 55 to 60 hours to ensure reliability.
3. Washing	Running tap water	3 hours to remove fixative in standard plastic cassettes (Eurocassette®). The absence of formaldehyde was confirmed by adding Schiff's reagent.
4. Dehydration	Ascending alcohol series	50% to 100% alcohol, 2-4 hours each step
5. Clearing	Xylene	2-4 hours total, in two portions
6. Embedding	Paraffin (Paraplast®)	60°C, 2 hours total. Finally, the joint capsule biopsies were individually hand-cast into 2 x 3 cm metal molds.
7. Sectioning	Sled microtome (Jung	4 µm thick sections

Step	Process	Details
	Histoslide 2000®)	
8. Mounting	Silanized slides (Histobond®)	Water bath stretching at 40°C
9. Adhesion	Custom adhesive	1:1 commercial glue and Poly-L-Lysine
10. Drying	Incubator	37-45°C for at least 12 hours
11. Deparaffinization	Xylene and descending alcohol series	Xylene (2x10 min), 100% Ethanol (2x3 min), 96% to 70% Ethanol (1x3 min each). Rehydration in distilled water for 1-2 minutes.

Table 3: Frequencies of canine stifle joint capsule samples and conditions among various breeds

Breed Group	Breed	Patellar Luxation (PL)	Cruciate Ligament Rupture (CCLR)	PL + CCLR	Control group	Sample Count (n)
Toy breeds	Chihuahua	10	0	0	1	11
	Chihuahua Mix	0	1	0	0	1
	Russkiy Toy	1	0	0	0	1
	Prager Rattler	2	0	0	0	2
Small breeds	Rehpinscher	1	0	0	0	1
	Rehpinscher Mix	1	0	0	0	1
	Yorkshire Terrier	5	0	1	0	6
	Yorkshire Terrier Mix	1	0	0	0	1
	Maltese	2	0	1	0	3
	Bolonka Zwetna	2	0	0	0	2
	Shih Tzu	0	1	0	0	1
	Pekingese	1	0	0	0	1
	Chinese Crested Dog	1	0	0	0	1
	Cavalier King Charles Spaniel	2	0	0	0	2
	Miniature Poodle	2	0	0	1	3
	Boston Terrier	1	0	0	0	1
	West Highland White Terrier	3	0	1	0	4
	Mixed Breed < 10 kg	6	0	0	1	7
Medium-Sized breeds	Shetland Sheepdog	1	0	0	0	1
	Shetland Sheepdog Mix	1	0	0	0	1
	French Bulldog	1	0	0	0	1
	Tibetan Terrier	1	0	0	0	1
	Tibetan Terrier Mix	0	1	0	0	1
	Beagle	0	1	0	0	1
	Border Collie	1	0	0	0	1
	Collie Mix	0	1	0	0	1
	Basset Fauve de Bretagne	1	0	0	0	1
	Shar Pei	2	0	0	0	2
	Podenco Canario Mix	1	0	0	0	1
	Mixed Breed 10 - 20 kg	5	1	1	0	7
Large breeds	German Shepherd Mix	1	0	0	0	1
	Boxer	0	2	0	0	2
	Akita Inu	1	0	0	0	1
	American Staffordshire Terrier	1	0	0	1	2

	Caucasian Ovcharka Mix	0	1	0	0	1
	Leonberger	0	1	0	0	1
	Great Dane	0	1	0	0	1
	Mixed Breed > 20 kg	1	0	0	0	1

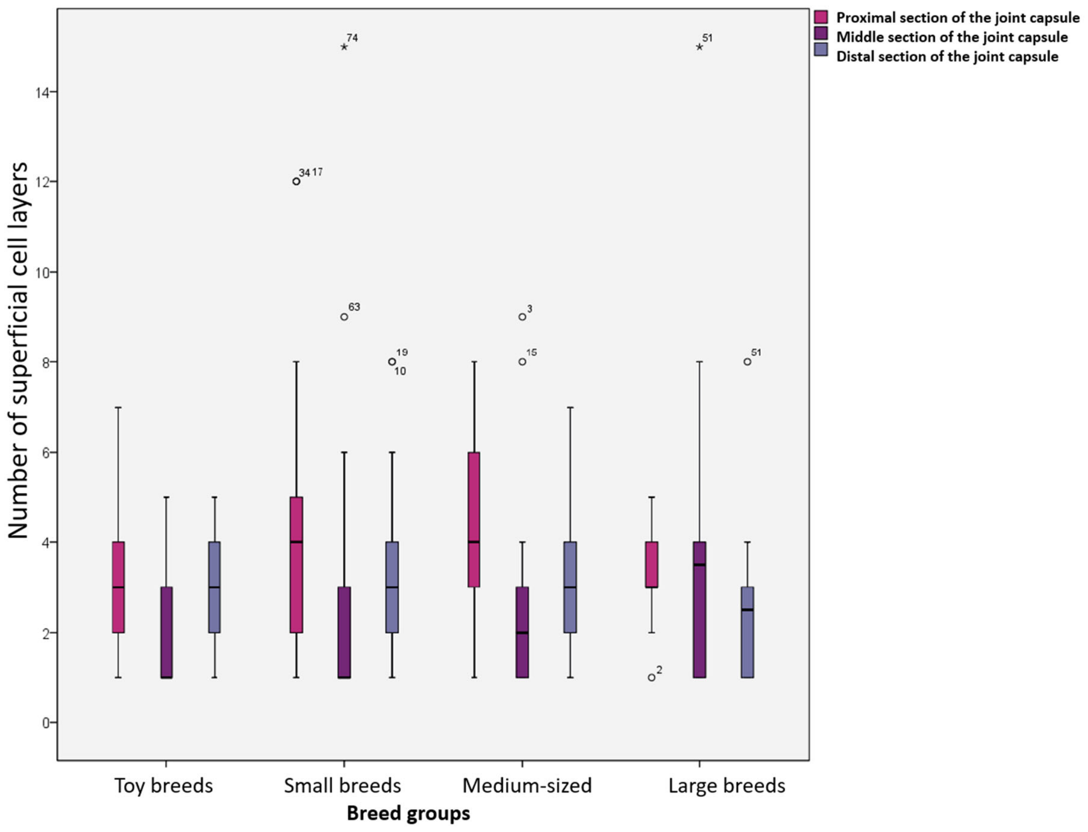

Figure 1: Number of superficial cell layers with distinction between the four breed groups in the different sections of the joint capsule.

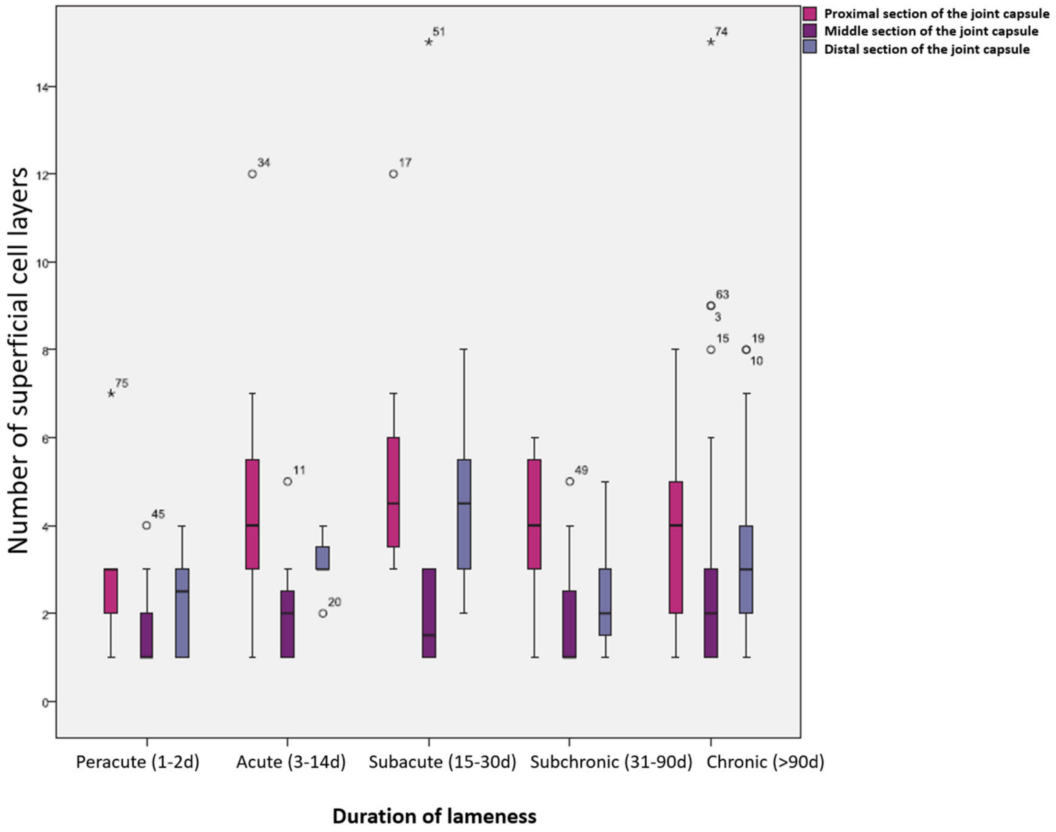

Figure 2: Number of surface cell layers in the three joint capsule sections according to duration of lameness

Table 4: Number of superficial cell layers in the three joint capsule sections by age

**Age in years**	**Number of cases**	**Number of superficial cell layers**		
		**Proximal section**	**Middle section**	**Distal section**
0,5	1	7	1	3
0,75	4	4	2,5	4
1,5	4	4,5	1,75	3
1,75	1	2	1	3
2	7	4	1,5	3
2,5	5	3,5	2	2,5
3	3	4,5	1,5	3,5
3,5	2	2,5	1	3
4	5	3	2,5	3,5
5	5	5	2	4

5,5	1	2	1	5
6	5	2,5	2	2,5
7	3	5	1	3
7,5	1	8	3	7
8,5	4	4,5	2	4
9	1	5	4	7
10,5	1	3	1	3

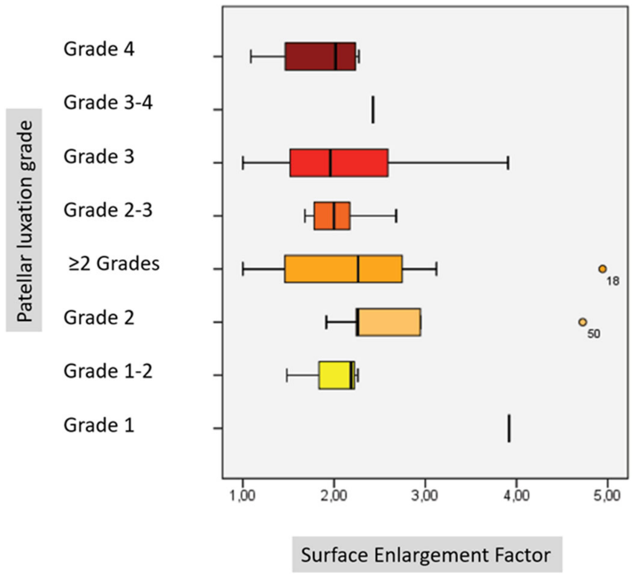

Figure 3: Surface enlargement factor values and patellar luxation grade.

Table 5: Progression forms of lameness with corresponding characteristics of surface enlargement factors

	**Surface enlargement factor**

Disease progression	N	Mean	Minimum	Maximum	Median	SD
Peracute (1 - 2 d)	8	2,60	1,09	4,86	2,65	1,17
Acute (3 - 14 d)	7	2,56	1,68	3,91	2,50	0,8
Subacute (15 - 30 d)	8	2,20	1,52	2,98	2,22	0,45
Subchronic (31 - 90 d)	7	2,92	1,00	4,94	3,10	1,37
Chronic (> 90 d)	26	2,46	1,14	4,73	2,30	0,86

Figure 4: Surface enlargement factors in disease courses

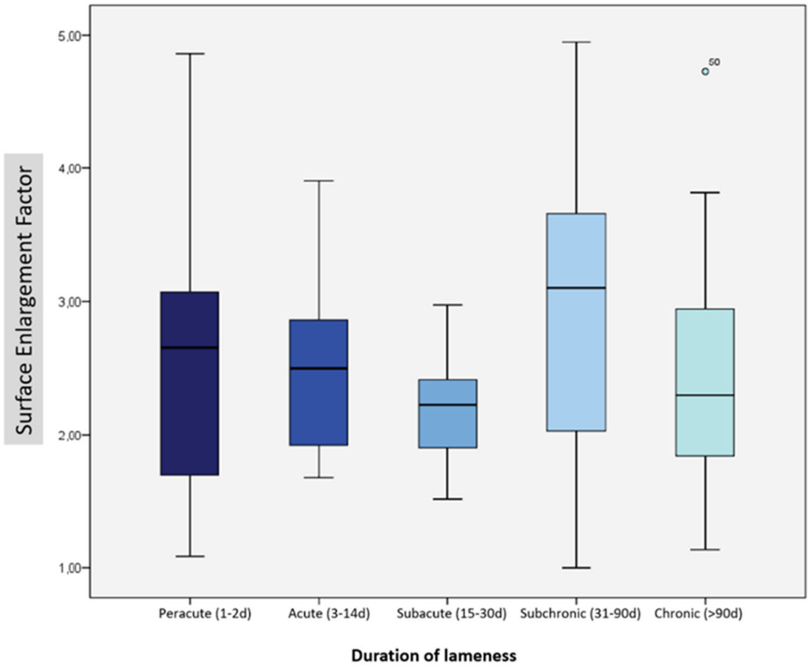

Table 6: Surface enlargement factor in the different breed groups

Breed groups	N	Surface Enlargement Factor				
		Mean	Minimum	Maximum	Median	SD
Toy breeds	15	2,17	1.09	3.57	2.19	0.60

Small breeds	31	2,39	1	4.94	2.14	0.95
Middle-sized breeds	14	2,52	1	4.73	2.3	0.99
Large breeds	10	2,29	1	3.82	2.12	1.01

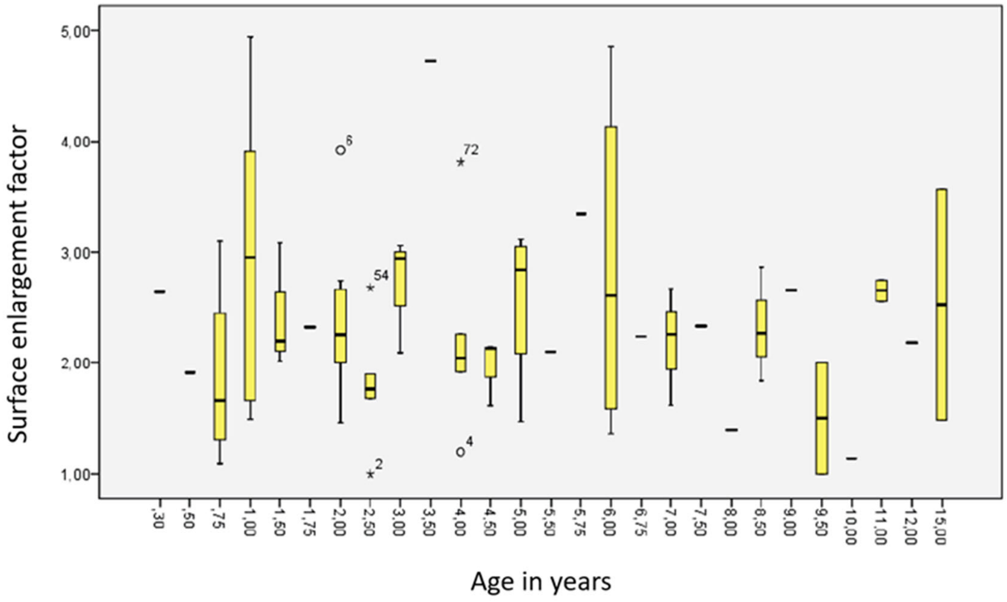

Figure 5: Surface enlargement factors and age of the patients

Table 7: Presence/absence of the middle section of the stratum subsynoviale in the four breed groups of the studied population

**Stratum subsynoviale**	**Breed groups**			
	Toy breeds	Small breeds	Middle-sized breeds	Large breeds

**present**	4 (26,7 %)	7 (20,6 %)	5 (26,3 %)	3 (30 %)
**absent**	11 (73,3 %)	27 (79,4 %)	14 (73,7 %)	7 (70 %)

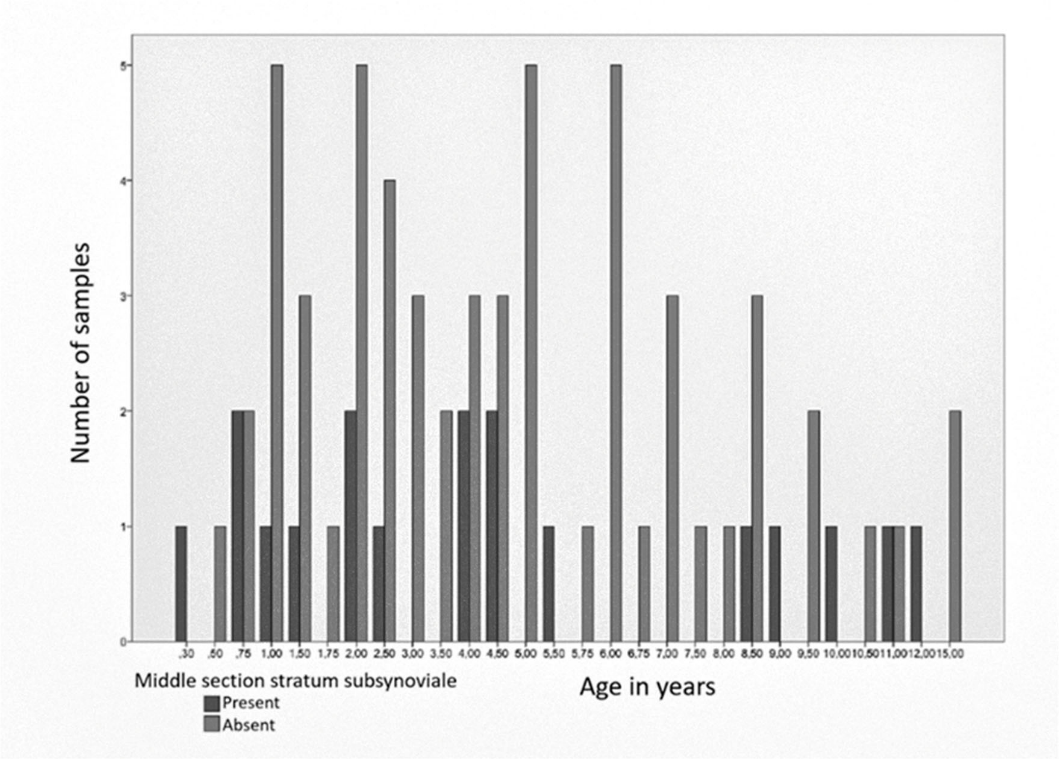

Figure 6- Frequencies of cases with present and absent middle section stratum subsynoviale by age groups in the studied population

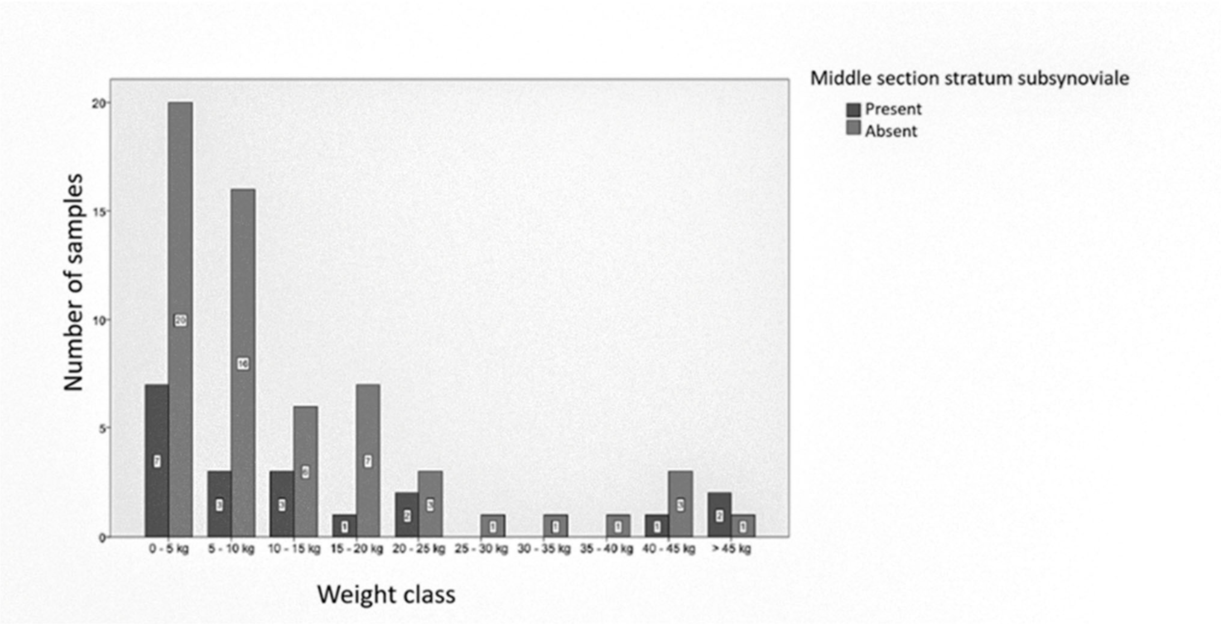

Figure 7: Frequencies of cases with (dark grey) and without (lighter grey) middle subsynovial layer across the ten weight classes of the examined sample

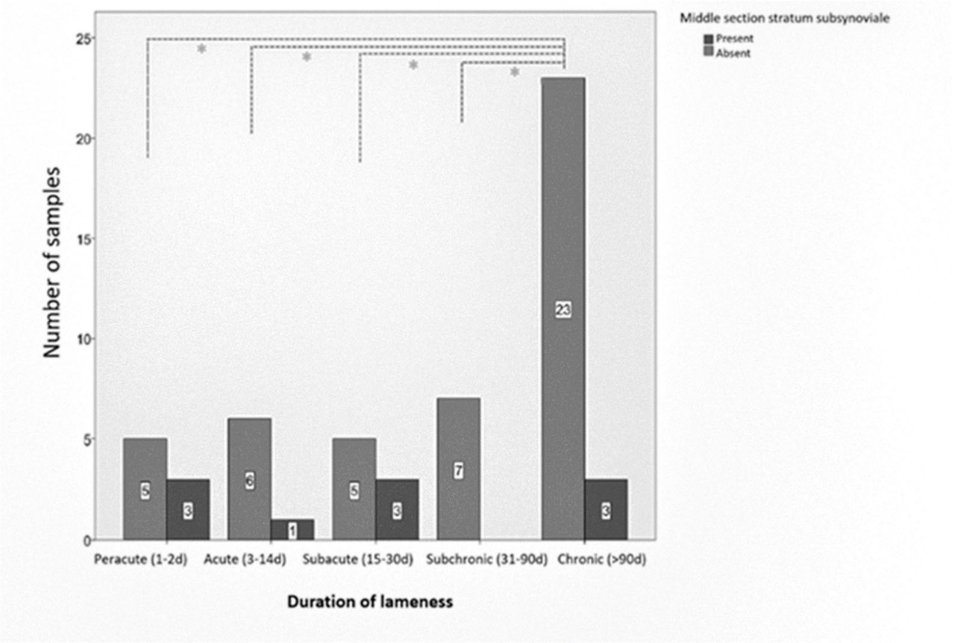

Figure 8: Frequencies of cases with and without subsynovial layer in the middle section of the stifle joint capsule in different stages of lameness. *Significance Level at $p < 0.05$.

Table 8- Joint capsule section and comparison of average thickness in the different breed groups

**Section / Layer**	**Breed Group Comparison**	**Sample Size (n)**	**Percentage Difference**	**P-Value (Bonferroni)**
Proximal section of stratum fibrosum	Toy dogs vs. Medium-sized dogs	15	42.70% thinner	0.049
Middle section of stratum fibrosum	Toy dogs vs. Medium-sized dogs	15	68.03% thinner	0.006
Distal section of stratum fibrosum	Toy dogs vs. Medium-sized dogs	15	49.02% thinner	0.048
Proximal section of stratum subsynoviale	Toy dogs vs. Large dogs	15	66.85% thinner	0.002
Proximal section of stratum subsynoviale	Small dogs vs. Large dogs	34	65.73% thinner	0.000
Middle section of stratum synoviale	Toy dogs vs. Large dogs	15	63.96% thinner	0.009
Middle section of stratum synoviale	Medium-sized dogs vs. Large dogs	19	53.76% thinner	0.021

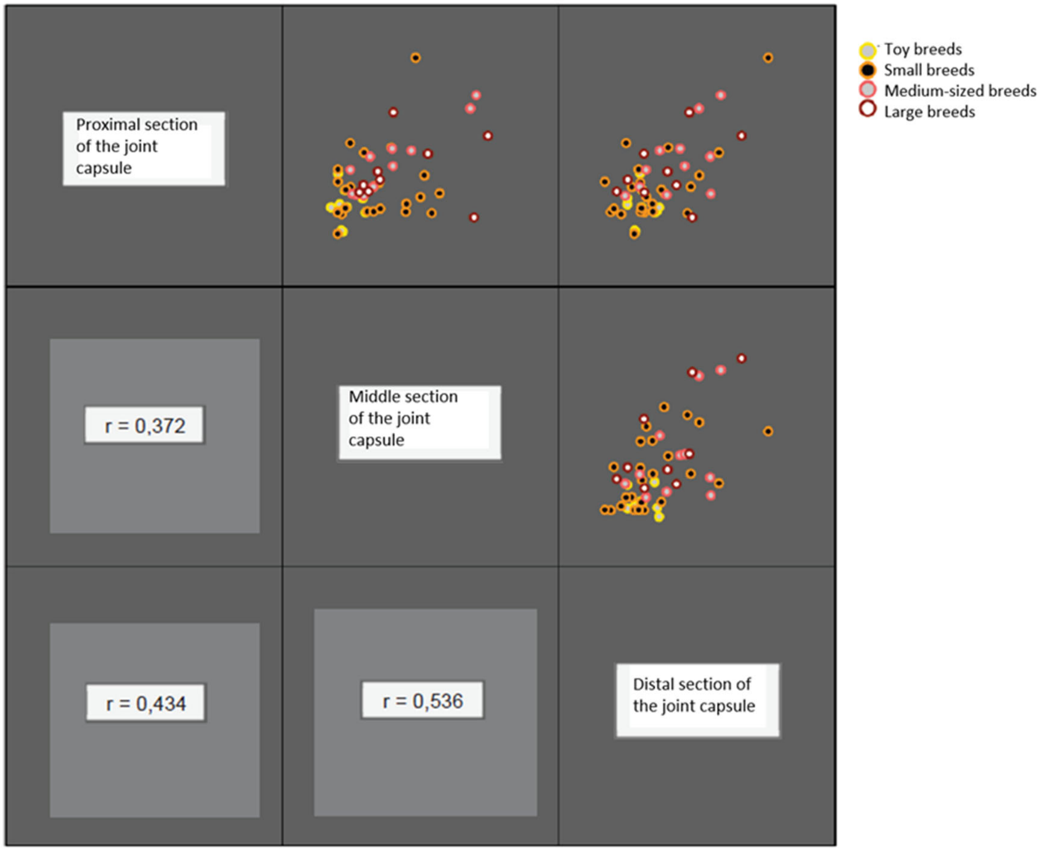

Figure 9: Correlation of the three joint capsule sections with each other, including the corresponding correlation coefficients r, highlighting the individual breed groups.

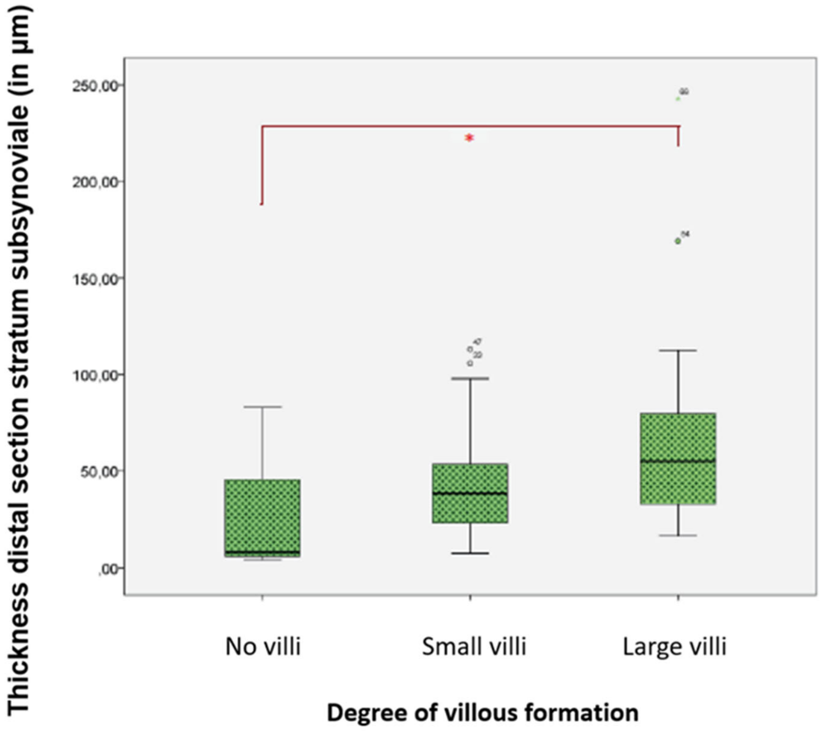

Figure 10: Thickness of subsynovial stratum at the distal section and the three degrees of villi formation. Significance (*) between No villi and large villi.
